# The impact of manual threshold selection in medical additive manufacturing

**DOI:** 10.1007/s11548-016-1490-4

**Published:** 2016-10-07

**Authors:** Maureen van Eijnatten, Juha Koivisto, Kalle Karhu, Tymour Forouzanfar, Jan Wolff

**Affiliations:** 1 Department of Oral and Maxillofacial Surgery/Oral Pathology, 3D Innovation Lab, VU University Medical Center, De Boelelaan 1118, 1081 HV Amsterdam, The Netherlands; 2 Research and Technology, Planmeca Ltd, Helsinki, Finland

**Keywords:** Additive manufacturing, Three-dimensional (3D)printing, Computed tomography (CT), Medical imaging, Segmentation, Thresholding

## Abstract

**Purpose:**

Medical additive manufacturing requires standard tessellation language (STL) models. Such models are commonly derived from computed tomography (CT) images using thresholding. Threshold selection can be performed manually or automatically. The aim of this study was to assess the impact of manual and default threshold selection on the reliability and accuracy of skull STL models using different CT technologies.

**Method:**

One female and one male human cadaver head were imaged using multi-detector row CT, dual-energy CT, and two cone-beam CT scanners. Four medical engineers manually thresholded the bony structures on all CT images. The lowest and highest selected mean threshold values and the default threshold value were used to generate skull STL models. Geometric variations between all manually thresholded STL models were calculated. Furthermore, in order to calculate the accuracy of the manually and default thresholded STL models, all STL models were superimposed on an optical scan of the dry female and male skulls (“gold standard”).

**Results:**

The intra- and inter-observer variability of the manual threshold selection was good (intra-class correlation coefficients >0.9). All engineers selected grey values closer to soft tissue to compensate for bone voids. Geometric variations between the manually thresholded STL models were 0.13 mm (multi-detector row CT), 0.59 mm (dual-energy CT), and 0.55 mm (cone-beam CT). All STL models demonstrated inaccuracies ranging from −0.8 to +1.1 mm (multi-detector row CT), −0.7 to +2.0 mm (dual-energy CT), and −2.3 to +4.8 mm (cone-beam CT).

**Conclusions:**

This study demonstrates that manual threshold selection results in better STL models than default thresholding. The use of dual-energy CT and cone-beam CT technology in its present form does not deliver reliable or accurate STL models for medical additive manufacturing. New approaches are required that are based on pattern recognition and machine learning algorithms.

## Introduction

Additive manufacturing (AM), also known as three-dimensional (3D) printing, refers to a process where a series of successive layers are laid down to create a 3D construct. AM combined with advanced medical imaging technologies such as computed tomography (CT) and magnetic resonance imaging (MRI) has resulted in a paradigm shift in medicine from traditional serial production to patient-specific constructs. This combination of technologies offers new possibilities for the fabrication of implants, saw guides and drill guides that are designed to meet the specific anatomical needs of patients [1].

**Fig. 1:**
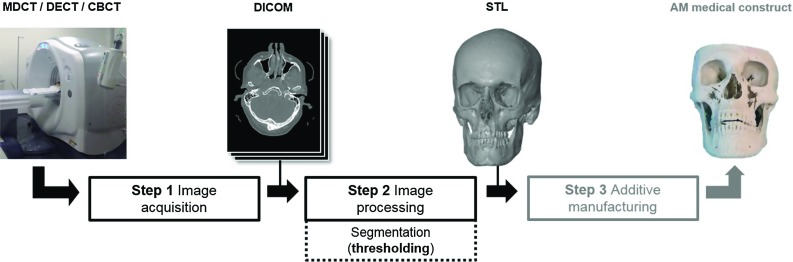
A schematic diagram of the three steps required to fabricate an AM medical construct

The three-step medical AM process begins with image acquisition (Fig. 1, Step 1), which is commonly performed using a multi-detector row computed tomography (MDCT) scanner. However, dual-energy computed tomography (DECT), which offers the possibility of acquiring CT images using two different X-ray spectra, is becoming more common in hospital environments [2]. Furthermore, cone-beam computed tomography (CBCT) is being increasingly used in dentistry and maxillofacial surgery due to its low costs and reduced radiation dose when compared with MDCT scanners [3].

Images acquired using CT technologies are commonly saved as Digital Imaging and Communications in Medicine (DICOM) files. These files contain voxels with grey values that are proportional to the attenuation coefficient in the corresponding volume of the patient. In MDCT, these grey values are scaled according to Hounsfield units (HU): air (−1000 HU), water (0 HU), and compact bone (+1000 HU). In CBCT technology, the degree of X-ray attenuation is scaled using grey values, hence voxel values [4]. CBCT grey values are often arbitrary and do not correspond to MDCT HU values [3, 5, 6]. Furthermore, a large variability in the grey values has been reported between different CBCT scanners [7, 8].

At present, medical AM requires the conversion of DICOM images into virtual 3D surface models that are commonly saved as standard tessellation language (STL) files (Fig. 1, Step 2). STL models are commonly used to design medical constructs using computer-aided design (CAD) software. The DICOM-to-STL conversion process requires the partitioning and hence the segmentation of voxels into different tissue types. The most common segmentation method used to date is thresholding. During the thresholding process, all voxels with a grey value that is equal or greater than a selected threshold value *t* are included in a segmented volume [9] using a binary mask $M_{x,y}$ (Eq. 1):

1 $$\begin{aligned} \hbox {M}_{\mathrm{x,y}} =\left\{ {\begin{array}{lllll} 0&{}\quad \hbox {I}_{\mathrm{x,y}} &{}<&{}t \\ 1&{}\quad \hbox {I}_{\mathrm{x,y}} &{}\ge &{}\hbox {t} \\ \end{array}},\right. \end{aligned}$$

where $\hbox {I}_{\mathrm{x,y}}$ denotes the grey value at coordinates *x* and *y*.

**Fig. 2:**
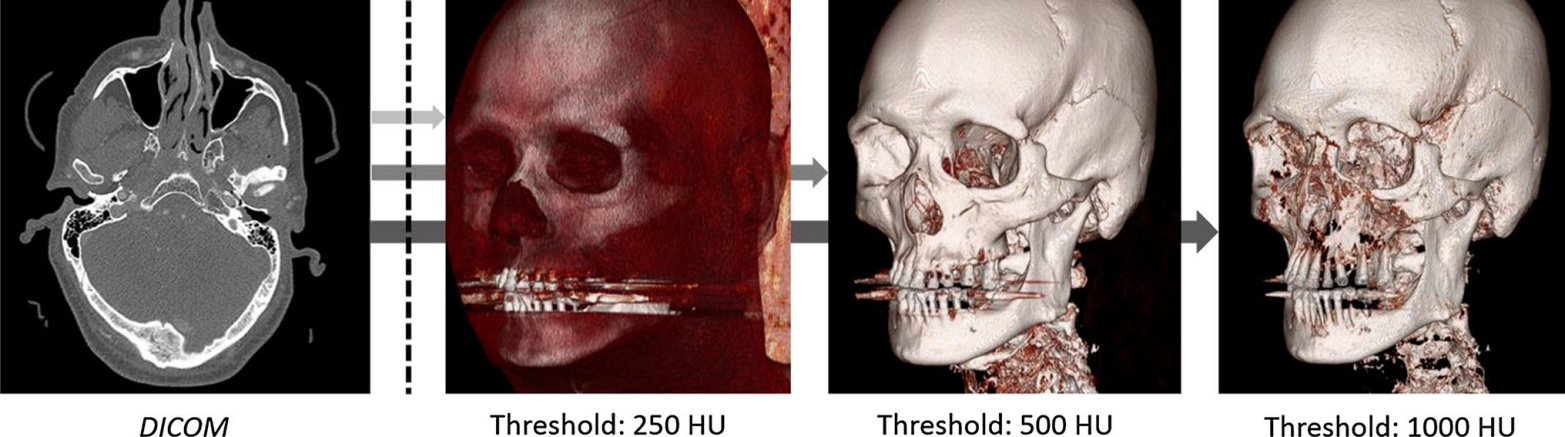
The effect of threshold selection on skull STL models

The medical image segmentation software packages available offer only a single, default threshold value for compact bone, soft tissue, and cartilage. However, these default values are often not optimized for all types of MDCT, DECT, and CBCT images and do not take into account the variations in grey values between different scanners [10]. Therefore, in most cases, manual threshold selection is necessary to acquire an optimal STL model. Threshold selection, however, still remains a subjective task [11], especially in the head area due to the plethora of complex bony geometries (Fig. 2). Furthermore, minor dislocations in the facial area can have an impact on patient function and aesthetic appearance.

At present, there is a paucity of the literature on threshold selection in the head area for medical purposes. Therefore, the aim of this study was to assess the impact of manual and default threshold selection on the reliability and accuracy of skull STL models acquired using different MDCT and CBCT technologies.

## Materials and methods

One female and one male human cadaver head were anonymously provided by the Department of Anatomy, VU University Medical Center Amsterdam, The Netherlands. The two heads were embedded in a novel embalming liquid “Fix for Life” [12] that produces near life-like cadavers. Ethical approval for this study was provided by the Medical Ethical Committee of the VU University Medical Center (Ref. 2016.401).

**Fig. 3:**
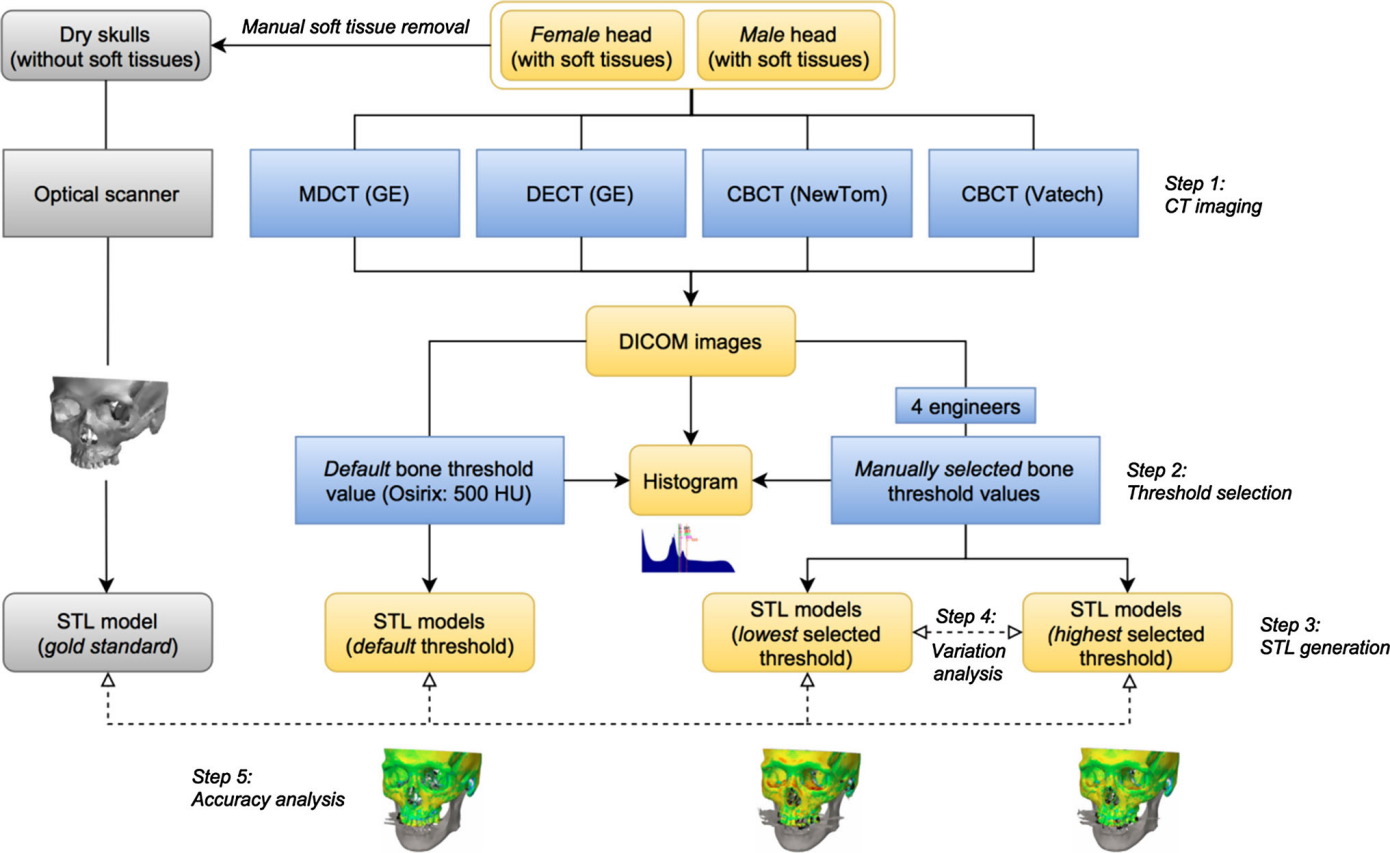
Outline of the study

The two “Fix for Life” cadaver heads were imaged using the following CT technologies: GE Discovery CT750 HD 64-slice MDCT (GE Healthcare, Little Chalfont, Buckinghamshire, UK), NewTom 5G CBCT (NewTom, Verona, Italy), and Vatech PaX Zenith 3D CBCT (Vatech, Fort Lee, USA) (Fig. 3, Step 1). The GE Discovery CT750 MDCT scanner was also operated using a dual-energy imaging mode (DECT). All scanners and image acquisition parameters are summarized in Table 1.

**Table 1:** Image acquisition parameters for all CT scans

	GE discovery CT750 HD 64-slice (MDCT)		GE discovery CT750 HD 64-slice (DECT)		NewTom 5G (CBCT)		Vatech PaX Zenith 3D (CBCT)	
	Female	Male	Female	Male	Female	Male	Female	Male
Tube voltage (kV)	120	120	80,140	80,140	110	110	115	115
Tube current (mA)	300	300	375	375	7	7	6	6
Exposure time (s)	0.638	0.912	0.912	0.699	10	10	24	24
Spacing between slices (mm)	0.312	0.312	0.312	0.312	n.a.	n.a.	n.a.	n.a.
Slices thickness (mm)	0.625	0.625	0.625	0.625	0.300	0.300	0.300	0.300
Number of voxels	512 $\times$ 512 $\times$ 767	512 $\times$ 512 $\times$ 919	512 $\times$ 512 $\times$ 767	512 $\times$ 512 $\times$ 919	610 $\times$ 610 $\times$ 539	610 $\times$ 610 $\times$ 541	800 $\times$ 800 $\times$ 632	800 $\times$ 800 $\times$ 632
Reconstruction kernel	Boneplus	Boneplus	Soft	Soft	Standard	Standard	n.a.	n.a.

After CT image acquisition, all DICOM files were imported into Osirix$^{{\circledR }}$ MD software (Osirix Foundation, Geneva, Switzerland). This software is FDA-cleared, CE-labelled for primary diagnostics, and is commonly used in medical AM. Osirix$^{{\circledR }}$ MD software provides options for both manual and default threshold selection.

Four medical engineers were subsequently requested to manually select the optimal threshold value for bone in order to create an accurate STL model of the female and male skull, hence facial bony structures (Fig. 3, Step 2). All four engineers were blinded for their own results and those of others. The manual threshold selection procedure was repeated after a six-week interval in order to determine the intra-observer variability and to calculate the mean threshold value. In addition, the inter-observer variability and intra-class correlation coefficients (ICC) were calculated using SPSS$^{{\circledR }}$ software (SPSS$^{{\circledR }}$ version 22, Chicago, IL, USA). ICC ranges between 0 and 1, with 0 corresponding to no agreement and 1 corresponding to complete agreement [13].

**Fig. 4:**
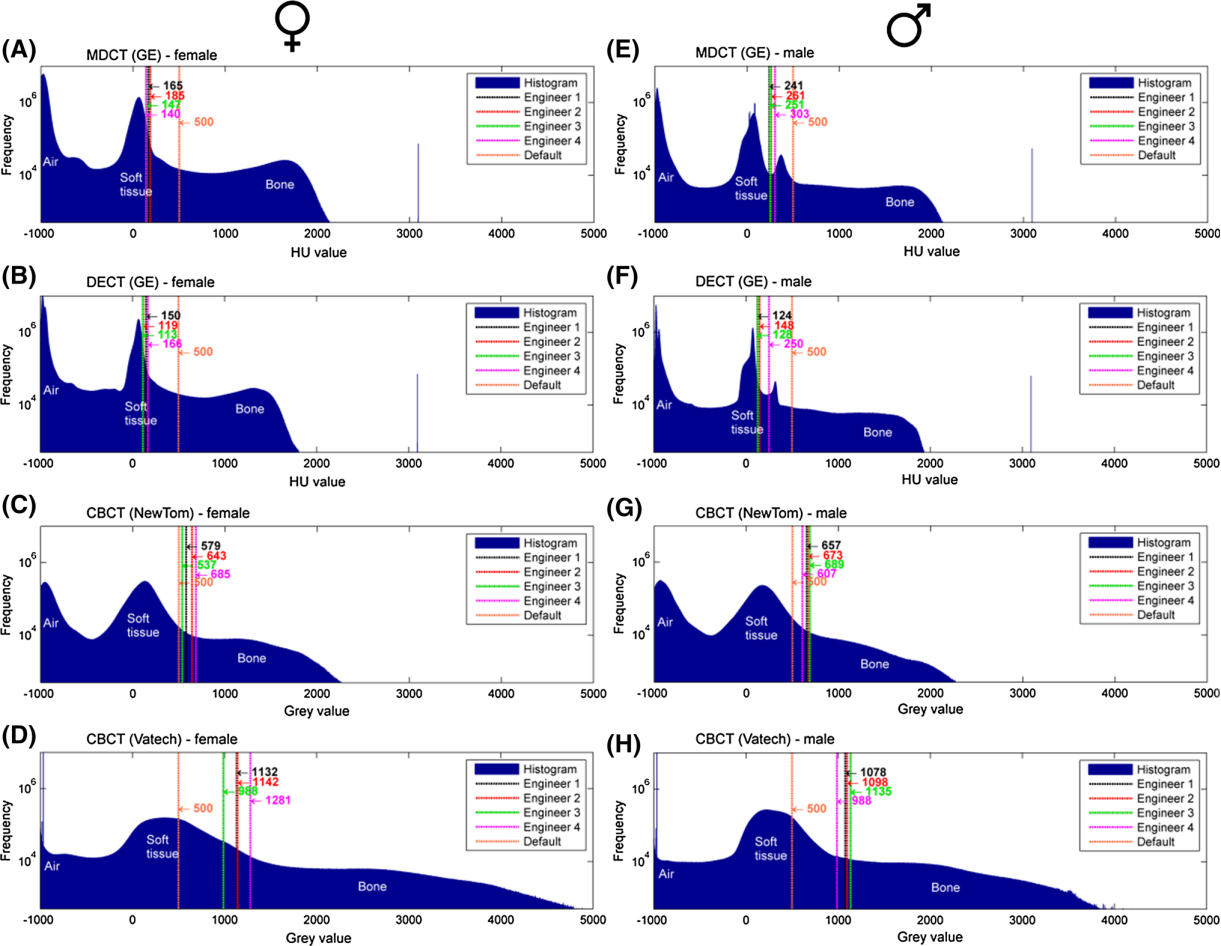
**a–h** The mean threshold values (HU) selected by four medical engineers and the pre-defined default threshold value (500 HU) are presented in histograms **a**–**h**. The y-axis of the histograms (frequencies) is set to a logarithmic scale

In order to graphically represent the distribution of grey values in the manually selected and default threshold values, histograms were plotted for each of the four CT scanners using MatLab$^{{\circledR }}$ software (MatLab v.2012, MathWorks, Natick, Massachusetts, USA) (Fig. 4). Only the highest and lowest mean selected threshold values presented on the eight histograms were used to generate STL models (Fig. 3, Step 3). The generated STL models were subsequently geometrically compared to each other using GOM Inspect$^{{\circledR }}$ software (GOM Inspect v8, GOM mbH, Braunschweig, Germany) in order to calculate the variations between the highest and lowest threshold STL models (Fig. 3, Step 4).

**Fig. 5:**
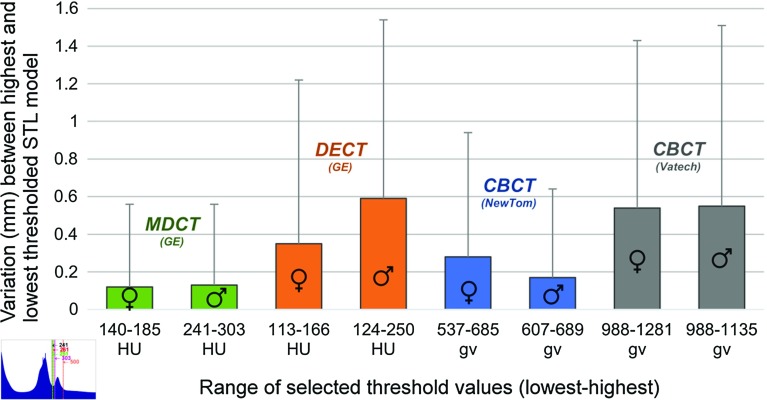
Geometric variations in mm between the highest and lowest thresholded STL models acquired using four different CT scanners (see also Fig. 4).

In a final step, all soft tissues were manually removed from the cadaver heads using standard dissection equipment (i.e., scrapers and scalpels) by a highly experienced technician at the Department of Anatomy. Manual removal was opted for since this procedure ensured minimal dimensional changes in the bony structures of the cadaver skulls [14]. The resulting dry female and male skulls were subsequently scanned using a GOM ATOS$^{\mathrm{TM}}$ III optical 3D scanner (GOM GmbH, Braunschweig, Germany) with an accuracy of <0.05 mm to acquire a “gold standard” STL model of the skulls (Fig. 3). These “gold standard” STL models were subsequently superimposed on the STL models generated using the highest and lowest manually selected and default threshold values in order to calculate the accuracy of each thresholded STL model (Fig. 3, Step 5).

**Fig. 6:**
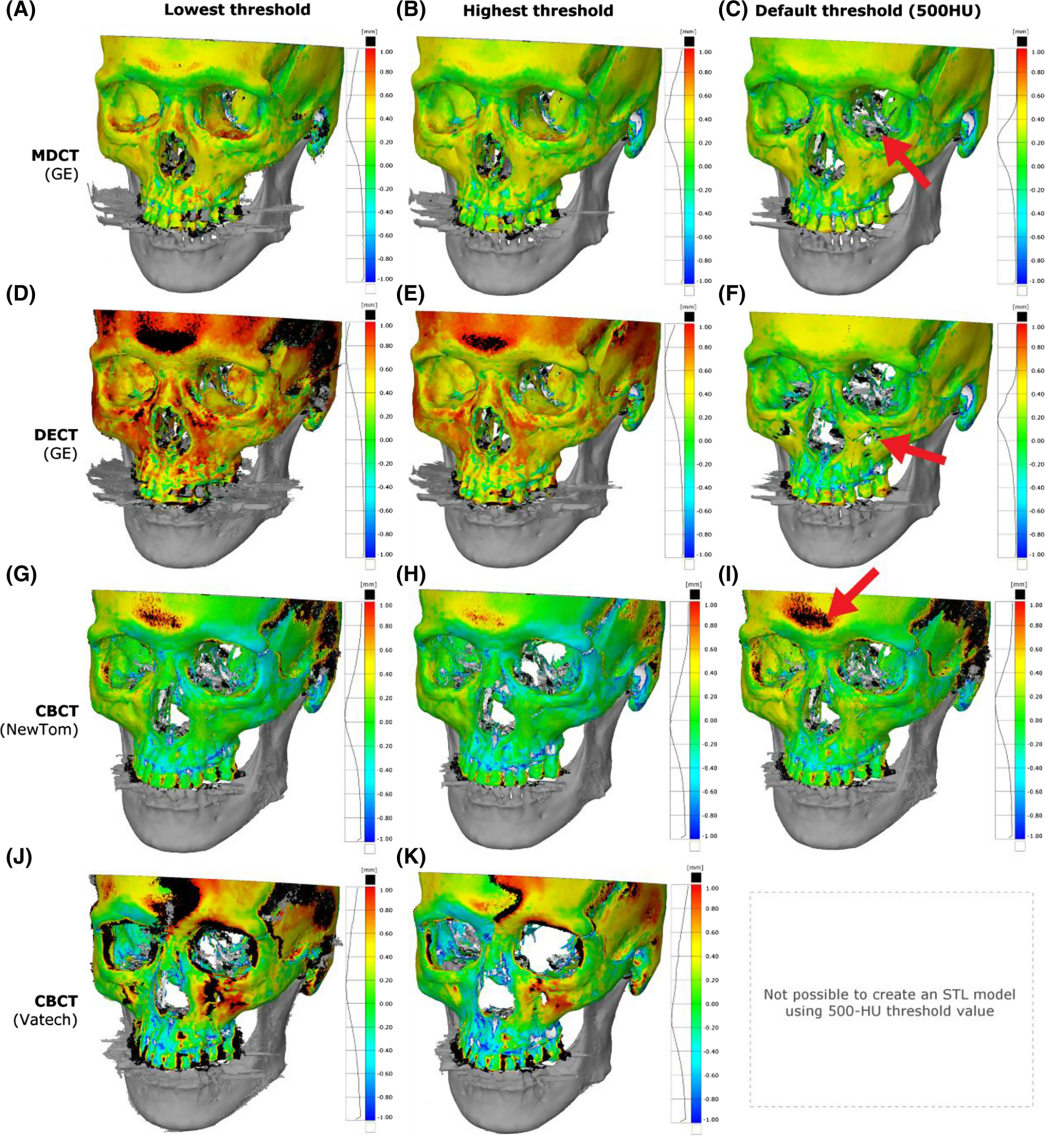
**(a–k)** Accuracy of all STL models of the female skull acquired using the lowest (*left*) and highest (*middle*) mean threshold value selected by the four engineers and the default threshold value of 500 HU (*right*). The arrows indicate missing data (**c**, **f**) or excessive noise (**i**) in the default threshold STL models

## Results

The intra- and inter-observer reliability results of all manually selected threshold values are presented in Table 2. All selected threshold values ranged from 113 to 303 HU for the MDCT and DECT technologies and from 537 to 1281 gv for the CBCT technologies (Fig. 4a–h). As shown in the histograms, all the selected threshold values differed from the default threshold value provided by Osirix MD$^{{\circledR }}$ software (500 HU). Furthermore, the geometric variations between the highest and lowest thresholded STL models were larger in the STL models derived from DECT and CBCT when compared with the MDCT-derived STL models (Fig. 5).

When compared to the “gold standard”, all manually and automatically thresholded STL models demonstrated inaccuracies ranging from −0.8 to +1.1 mm, −0.7 to +2.0 mm, and −2.3 to +4.8 mm for all STL models derived from MDCT, DECT, and CBCT, respectively (Fig. 6a–k). The male skull presented comparable accuracies to those observed on the female skull. The MDCT- and DECT-derived STL models acquired using the default threshold value demonstrated the highest loss of bone HU values (Fig. 6c, f). The NewTom CBCT-derived STL model acquired using the default threshold value (500 HU) provided by Osirix MD software resulted in an increase in artefacts and noise (Fig. 6i). The Vatech CBCT DICOM images did not allow the creation of an STL model using the 500-HU default threshold value since the grey values were not scaled to HU values (Fig. 4d, h).

**Table 2:** Intra- and inter-observer variability of manual threshold selection by four medical engineers on CT images of a female and a male cadaver head

Intra-observer variability		Inter-observer variability between the engineers			
Intra-class correlation coefficient (ICC)			ICC	ICC	ICC
			Engineer 2	Engineer 3	Engineer 4
Cadaver head	Female/male		Female/male	Female/male	Female/male
Engineer 1	0.999/0.997	Engineer 1	0.994/0.988	0.980/0.987	0.970/0.954
Engineer 2	0.995/0.995	Engineer 2		0.978/0.998	0.961/0.931
Engineer 3	0.992/0.999	Engineer 3			0.914/0.917
Engineer 4	0.969/0.989	Engineer 4			

## Discussion

To date, thresholding is the most commonly used segmentation method in medical AM. However, accurate bone segmentation often requires manual threshold selection, which still remains a subjective task. Moreover, recent studies suggest that the majority of inaccuracies that occur during the medical AM process are introduced during the image acquisition and image processing phases, rather than during the manufacturing, i.e., the 3D printing process itself [15–17]. Such inaccuracies can markedly influence the resulting STL model (see Fig. 6) and subsequently lead to ill-fitting AM implants [18]. Therefore, the aim of the present study was to assess the impact of manual and automatic default threshold selection on the reliability and accuracy of skull STL models.

**Fig. 7:**
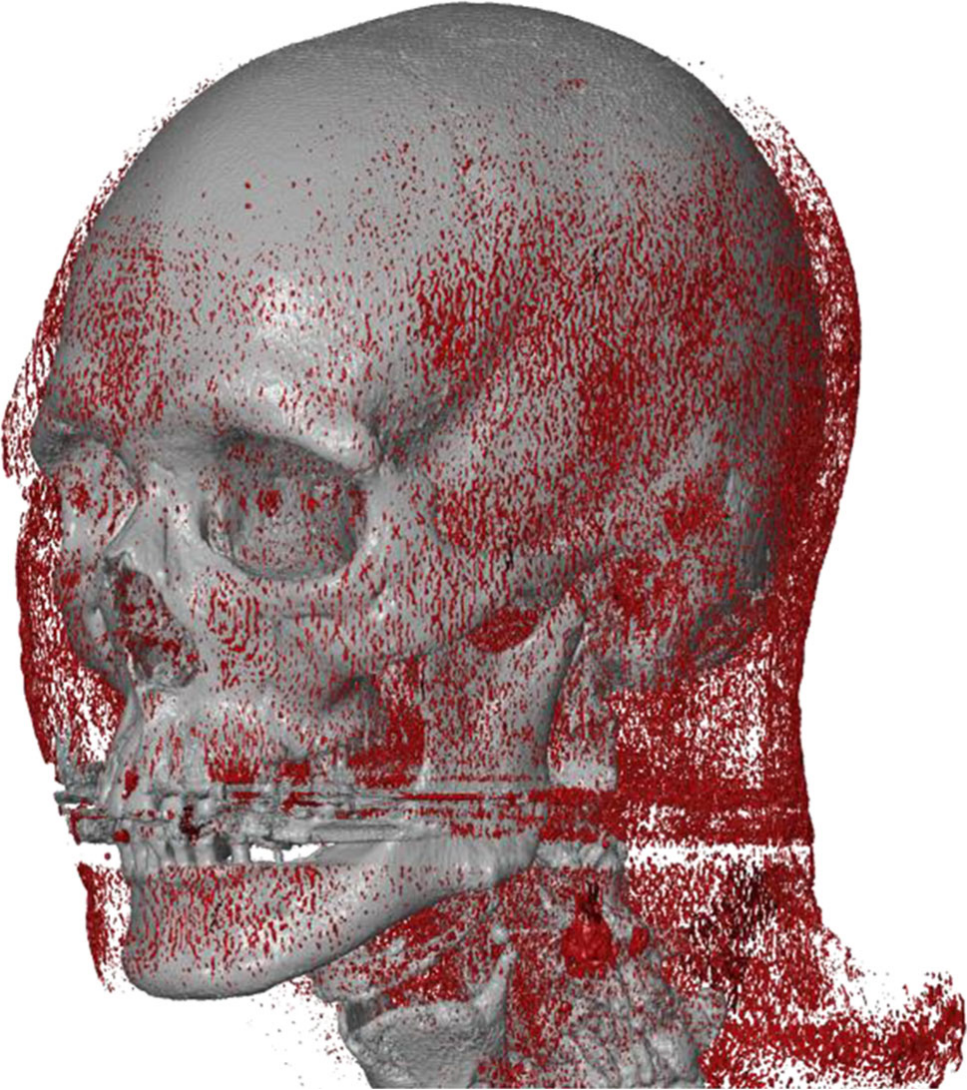
MDCT-derived low-threshold STL model of the female cadaver skull (*grey*) with disjointed “soft-tissue” structures (*red*)

In the present study, all threshold values selected by the four engineers demonstrated a good intra-observer reliability (ICC > 0.9). Furthermore, the inter-observer reliability was also good (ICC > 0.9), as shown in Table 2. Interestingly, all engineers that were blinded during the experiment selected threshold values for bone that were very close to the grey values of soft tissues (Fig. 4). This resulted in small disjointed structures in the STL model (marked red in Fig. 7) that represent the transition from bone into soft tissue grey values. Such disjointed “soft-tissue” structures can be manually removed during STL post-processing [19]. All engineers purposely selected the “soft tissue” threshold values during bone segmentation in order to incorporate the maximum number of bone-specific grey values. These grey values are allocated to voxels that represent different tissues during the CT image reconstruction process. However, during this process, voxels on the bone-to-soft tissue boundaries that are partially filled with soft tissue are commonly assigned a lower grey value than bone. This phenomenon is coined the partial volume effect (PVE) [20]. As a consequence of the PVE, voxels may be erroneously allocated to “soft tissue” instead of “bone”, resulting in data loss and hence bone voids in the STL model (Fig. 6). Therefore, engineers should be aware of this phenomenon since data loss can lead to large inaccuracies in individualized printed medical constructs [18, 20].

Another major finding in this study was the difference between the MDCT and CBCT DICOM files that were used to construct STL models (Fig. 4). One explanation for this phenomenon is the inherent difference between these technologies. CBCT technology is typically more heavily affected by image noise and distortions due to the “cone-beam” geometry of the X-ray beam [21, 22]. In CBCT, the simultaneously irradiated area is typically larger than in MDCT technology. This causes increased scatter levels and results in cupping, reduced contrast, and other scatter-induced artefacts in the reconstructed image. In addition, CBCT images are more subject to cone-beam artefacts due to the large cone-beam angle and the imaging geometry comprising a single focal plane. The cone-beam artefacts result from violating Tuy’s sufficiency condition [23] that requires that each plane intersecting a region of interest must intersect the focal trajectory, i.e., the path defining the radiation source position during the imaging. The embodiments of cone-beam artefacts are dependent on the reconstruction algorithm and the imaging geometry. Typical cone-beam artefacts include the elongation of structures in the axial direction and negative undershoots at sharp edges in the transaxial planes [24]. In CBCT, the focal trajectory consists of a single planar circle or arc that results in a violation of Tuy’s sufficiency condition in all regions outside the focal plane. The resulting cone-beam artefacts are more pronounced the further away the region of interest is from the focal plane. In MDCT, the volume that satisfies Tuy’s sufficiency condition is notably larger due to the helical nature of the focal trajectory.

The presence of artefacts makes the segmentation and hence the thresholding of bone-specific grey values in CBCT images more cumbersome [25]. This subsequently leads to a larger variation in manually selected threshold values for CBCT images (Fig. 4) and to the larger geometric variations of up to 0.55 mm in CBCT-derived STL models observed in this study (Fig. 5). DECT-derived STL models demonstrated geometric variations of up to 0.59 mm (Fig. 5). As a consequence of these geometric variations in STL models, the use of DECT and CBCT technology in its present form does not deliver reproducible STL models for medical AM. Therefore, the authors of this study suggest that only MDCT technology should be used for AM applications because of the lower variability (0.13 mm, see Fig.  5) and higher accuracy (Fig. 6) of the technology.

The present study demonstrates that the “human factor”, i.e., the medical engineer, influences the outcome of the segmentation process. Moreover, no single bone threshold value is applicable for all facial bones. The authors of this study therefore recommend the use of individual threshold values for each anatomical buttress. Recently, attempts have been made to develop novel segmentation algorithms using multi-thresholding [26], adaptive thresholding [11], and semi-automatic region growing [27]. However, these algorithms are still in an early stage of development [28] and do not take the inherent differences between MDCT and CBCT technologies into account. Future research should therefore focus on developing novel medical image segmentation software that is suitable for different CT imaging modalities. Furthermore, new approaches should be developed using pattern recognition and machine learning algorithms.

## Conclusion

This study shows that manual threshold selection results in better skull STL models than default thresholding since all the medical engineers in our study selected grey values closer to soft tissue to compensate for bone voids. Our study also showed that MDCT-derived STL models offer the lowest variability and highest accuracy, whilst the use of DECT and CBCT technology in its present form does not deliver reliable STL models for medical AM. New approaches based on pattern recognition and machine learning algorithms are required.
